# Combined Effect of Ultrasound Stimulations and Autoclaving on the Enhancement of Antibacterial Activity of ZnO and SiO_2_/ZnO Nanoparticles

**DOI:** 10.3390/nano8030129

**Published:** 2018-02-25

**Authors:** Hajer Rokbani, France Daigle, Abdellah Ajji

**Affiliations:** 1 3SPack, CREPEC, Department of Chemical Engineering, Polytechnique Montréal, P.O. Box 6079, Station Centre-Ville, Montreal, QC H3C 3A7, Canada; hajer.rokbani@polymtl.ca; 2 Department of Microbiology, Infectiology and Immunology, Pavillon Roger-Gaudry, Université de Montréal, P.O. Box 6128, Station Centre-ville, Montréal, QC H3C 3J7, Canada; france.daigle@umontreal.ca

**Keywords:** poly (lactic acid), ZnO nanoparticles, electrospinning process, antibacterial properties, *E. coli*

## Abstract

This study investigates the antibacterial activity (ABA) of suspensions of pure ZnO nanoparticles (ZnO-NPs) and mesoporous silica doped with ZnO (ZnO-UVM7), as well as electrospun nanofibers containing those nanoparticles. The minimum inhibitory concentration (MIC) and minimum bactericidal concentration (MBC) of these two materials were also determined under the same conditions. The results showed a concentration-dependent effect of antibacterial nanoparticles on the viability of *Escherichia coli* (*E. coli*). Moreover, the combination of the stimulations and sterilization considerably enhanced the antimicrobial activity (AMA) of the ZnO suspensions. Poly (lactic acid) (PLA) solutions in 2,2,2-trifluoroethanol (TFE) were mixed with different contents of nanoparticles and spun into nonwoven mats by the electrospinning process. The morphology of the mats was analyzed by scanning electron microscopy (SEM). The amount of nanoparticles contained in the mats was determined by thermogravimetric analysis (TGA). The obtained PLA-based mats showed a fibrous morphology, with an average diameter ranging from 350 to 450 nm, a porosity above 85%, but with the nanoparticles agglomeration on their surface. TGA analysis showed that the loss of ZnO-NPs increased with the increase of ZnO-NPs content in the PLA solutions and reached 79% for 1 wt % of ZnO-NPs, which was mainly due to the aggregation of nanoparticles in solution. The ABA of the obtained PLA mats was evaluated by the dynamic method according to the ASTM standard E2149. The results showed that, above an optimal concentration, the nanoparticle agglomeration reduced the antimicrobial efficiency of PLA mats. These mats have potential features for use as antimicrobial food packaging material.

## 1. Introduction

The development of antimicrobial packaging materials based on poly (lactic acid) (PLA), is growing continuously, with major focuses on enhancing food safety and quality and, concurrently, exploring alternatives to synthetic polymers made from petrochemicals that are less environmentally friendly [1]. Among the various aliphatic degradable polyesters, PLA has been considered as one of the most interesting and promising biodegradable materials [2]. Since it has been classified as “generally recognized as safe” (GRAS), PLA has been approved for use in food packaging, including direct-contact applications [3]. Moreover, it is suitable for producing thin and uniform electrospun nanofibers and can be dissolved in many common solvents [2,4]. ZnO-NPs are considered as antimicrobial agents that can compete favorably with silver nanoparticles, especially because of their simple and inexpensive synthesis, greater efficiency and cost-effectiveness, lower levels of toxicity [5,6], as well as their selective toxicity toward a wide range of both Gram-positive and Gram-negative bacteria, including major food-borne organisms such as *Escherichia coli, Listeria* and *Staphylococcus aureus* [7,8,9,10]. As inorganic antibacterial agents, ZnO-NPs have better stability at high temperatures and pressures, and longer shelf life than the organic ones [8,11,12]. Moreover, they show an ABA without photo-activation compared to TiO_2_ [7,10,11]. The main potential food application of ZnO-NPs is as an antimicrobial agent in food packaging materials [13,14]. When incorporated into antimicrobial packaging, ZnO-NPs can also improve the properties of the packaging material, such as mechanical strength, barrier properties, and stability [8,15]. Proper incorporation of ZnO-NPs into packaging materials will favor interaction with foodborne pathogens and act as bacteriostatic or bactericidal agents onto the food surface where bacteria reside, halting their growth and, thus, preventing food from spoilage [16].

Polymeric nanofibers containing antimicrobial nanoparticles can exhibit several advantages compared to standard organic compound-loaded polymeric analogs such as higher thermal stability, enhanced mechanical performance or biocompatibility, depending on the chemical nature of nanoparticles. Electrospinning is one of the most common methods of nanofiber formation for life science, protective clothing, filters, sensors, tissue engineering, drug delivery systems, and other applications. Synthetic and natural polymers, as well as their blends and composites with proper nanoparticles, are used in the electrospinning process to form nano- and submicron fibers with architecture and properties suitable for appropriate applications. Understanding of electrospinning process parameters enables tailoring of the electrospun nanofibers’ morphology, internal structure, and properties to their respective applications [17]. ZnO-NPs have been exploited for the development of several antibacterial fibrous materials based on biodegradable polymers. ZnO-NPs (100 nm) were incorporated into nanofibers based on poly (vinyl alcohol) (PVA) and sodium alginate and showed a substantial antibacterial activity (ABA) against *E. coli* and *S. aureus*, which was proportional to nanoparticles concentration [18]. Augustine and co-workers [19] found that concentrations higher than 5 wt % ZnO-NPs incorporated in polycaprolactone (PCL) nanofibers was able to inhibit the proliferation of *E. coli* and *S. aureus*, which makes them promising as antibacterial biodegradable scaffolds [19]. In a recent work, they proved that PCL/ZnO nanofibers with a concentration below 4 wt % ZnO-NPs, were efficient to heal wounds on the skin of animals without showing inflammation of tissues [20]. Virovska's group [21] combined the electrospinning and electrospraying processes to generate a PLA and ZnO non-woven textile that was active against *S. aureus*, but the ZnO content (45 wt %) used was considered unsuitable for adhesion and proliferation of tissue cells [21]. Rodriguez-Tobias and co-workers [22,23] noticed through two different studies that simultaneous electrospinning of PLA solutions with electrospraying of ZnO dispersions, as well as the use of ZnO-graft-PLA nanoparticles, provided better antibacterial performance than the PLA-based mats filled with pure ZnO-NPs and developed by the electrospinning process. The incorporation of ZnO-graft-PLA nanoparticles improved the nanoparticles’ dispersion within the PLA fibers and, consequently, enhanced their antibacterial (*E. coli* and *S. aureus*) and mechanical properties [22]. When electrospinning/electrospraying was used with at least 1 wt % of ZnO-NPs, the growth inhibition of *S. aureus* was around 94%, while simple electrospun mats did not inhibit the bacterium growth for the same ZnO concentration. Additionally, it was noticed that *E. coli* are less sensitive than *S. aureus* to the presence of nanoparticles [23]. 

The ABA between an antimicrobial agent dispersed in the packaging material and the food product can be achieved by either direct contact using a non-migratory system or by indirect contact using a volatile antimicrobial releasing system. Many reports have shown that the ABA of ZnO-NPs is size-dependent [7,9,10,24,25,26] and concentration-dependent [9,10,12,25,26,27,28,29,30,31,32]; in this way the antimicrobial activity (AMA) of ZnO-NPs on *E. coli* and *S. aureus* has been promoted by a decrease in particle size and an increase in concentration. Commonly-accepted mechanisms of antibacterial action of ZnO-NPs are the formation of reactive oxygen species (ROS) [7,27,28,33], the interaction of nanoparticles with bacteria [9,34,35,36], and the release of Zn^2+^ ions [24,37]. For ROS, whose amount generated should increase with the concentration of ZnO-NPs [7,24], they are supposed to cause the oxidation of the lipid membrane of the cell wall of *E. coli*. The direct interaction between ZnO-NPs and the surface of the cells affects the permeability of the membrane [9,36] allows the internalization of NPs [34] and induces oxidative stress in bacteria cells [38]. The toxicity of ZnO-NPs could result from the solubility of Zn^2+^ ions in the medium of micro-organisms [8,15].

The first objective of this research was to determine, for the first time, the combined influence of ultrasound and sterilization on inhibiting *E. coli* functions by the ZnO-NPs. To the best of our knowledge, the combinatory antibacterial effect of autoclaving, ultrasound stimulations, and ZnO-NPs on *E. coli* has not been studied previously. The only study that approached the impact of ultrasound stimulations on the antibacterial effect of ZnO-NPs suspensions has treated the suspensions containing both the bacteria cells and ZnO-NPs [39], whereas, in the present study, the nanoparticle suspensions were either sonicated and/or autoclaved before their inoculation. Seil's group study [39] focused on the effect of ultrasound on the physical interaction between the nanoparticles and the bacterial membrane only. These interactions may be enhanced due to nanoparticles dissociation and increased nanoparticles penetration into the cell membrane. However, in the present study, we focused on how the pretreatments (ultrasound-autoclaving) of ZnO-NPs suspensions can modify the nanoparticles’ physical state (agglomeration-dissociation) before any bacterial inoculation, and its effect on the ABA of their suspensions. The effect of these pretreatments was also extended to the minimum inhibitory concentration (MIC) and minimum bactericidal concentration (MBC) of the nanoparticle suspensions. To the best of our knowledge, the MIC and MBC of ZnO suspensions were estimated using different approaches [16,37,40,41] but none of them have tackled the effect of pretreatments. A comparative study was also performed on ZnO and silica nanoparticles doped with ZnO (ZnO-UVM-7) material for all the experiments described above. As a second objective, the ABA of PLA-based nanofibers loaded with ZnO and ZnO-UVM-7 materials was studied. These materials may be good candidates for antimicrobial food packaging material.

## 2. Results 

### 2.1. Effect of the Combined Effect of Sterilization and Ultrasound Stimulations on the ABA of Zinc Oxide Suspensions

The objective of these experiments was to determine, for the first time, the combined effect of sterilization, ultrasound stimulations and ZnO-NPs on *E. coli* ABA. In order to examine the effect of each parameter, as well as their combined effect, four different groups of experiments were conducted, as mentioned in the experimental section.

**Figure 1:**
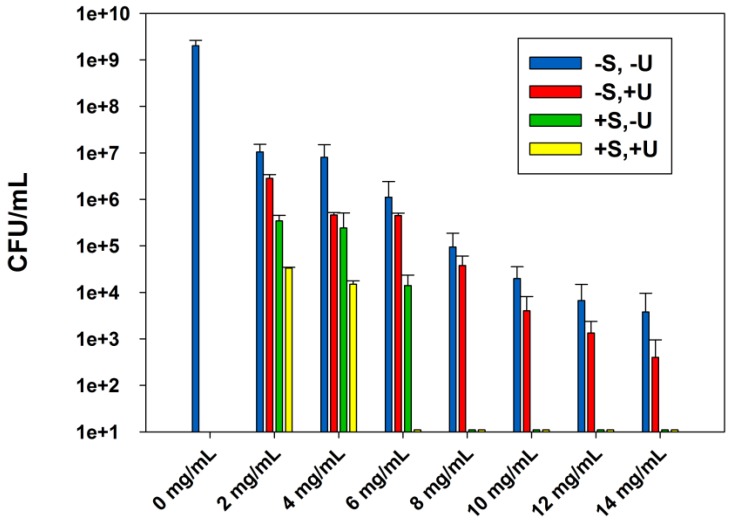
The effect of sterilization and ultrasound stimulations on the ABA of ZnO-NPs, in LB suspensions against *E. coli* (DH5α), after incubation at 37 °C for 24 h. Data are shown with mean values and standard errors of bacterial counts.

Figure 1 represents the effect of sterilization and ultrasound stimulations on the ABA of ZnO-NPs for concentrations from 0 to 14 mg/mL. For the first group of experiments where ZnO-NPs were used without any treatment, the results show that the increase of ZnO-NPs concentration in suspension from 2 to 14 mg/mL decreased *E. coli* growth gradually. The rise in ZnO-NP content was able to progressively increase the inhibitory effect of the *E. coli* growth, but a complete inhibition was not achieved even for the highest concentration of ZnO-NPs (14 mg/mL). At this concentration, ZnO-NPs were able to reduce the bacterial growth by 5 log CFU/mL. When the ZnO suspensions were sonicated before their inoculation, we observed the same trend on the inhibition of the *E. coli* growth. The ABA of ZnO-NPs was enhanced as their concentration in suspensions was increased. At 2 mg/mL, ZnO-NPs reduced *E. coli* growth from 2 × 10^9^ (Control) to 1 × 10^7^ and 2 × 10^6^ CFU/mL for the non-treated and the sonicated ZnO-NPs suspensions, respectively. At 4 mg/mL the sonicated ZnO-NPs reduced the *E. coli* population to 4 × 10^5^ CFU/mL, whereas the untreated ones inhibited it to 8 × 10^6^ CFU/mL. For the third group of experiments, the ZnO-NPs suspensions were autoclaved before their inoculation. In this case, the three first concentrations of ZnO-NPs were able to decrease the bacterial growth gradually, starting with 4 logs CFU/mL reduction for the lowest one. At a concentration of 8 mg/mL, the *E. coli* growth was totally inhibited. For the last group of experiments, ZnO suspensions were autoclaved and sonicated before their contamination with *E. coli*. The result showed that 2 and 4 mg/mL of ZnO-NPs were able to reduce the *E. coli* growth by four orders of magnitude, from 2 × 10^9^ (Control) to 3 × 10^4^ and 1 × 10^4^ CFU/mL, respectively. A concentration of 6 mg/mL was able to completely inhibit the *E. coli* growth.

**Figure 2:**
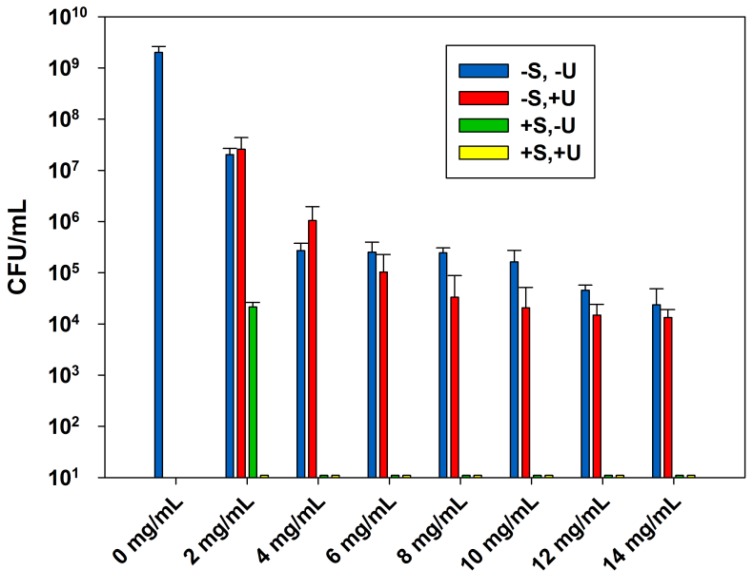
The effect of sterilization and ultrasound stimulations on the ABA of ZnO-UVM-7 (Si/Zn = 5) material, LB suspensions against *E. coli* (DH5α), after incubation at 37 °C for 24 h. Data are shown with mean values and standard errors of bacterial counts.

Figure 2 represents the effect of sterilization and ultrasound stimulations on the ABA of ZnO-UVM-7 (Si/Zn = 5) material for concentrations from 0 to 14 mg/mL. This antibacterial material has been tested in the same way as ZnO-NPs above to evaluate the effect of sterilization and ultrasound stimulations on its ABA. For the first groups of experiments, the increase of ZnO-UVM-7 content decreased progressively the *E. coli* population. A concentration of 2 mg/mL reduced the *E. coli* population by two orders of magnitude, compared to control suspensions with no powders. At 4 mg/mL, the bacterial growth was reduced to 2 × 10^5^ CFU/mL and remained approximately in the same range for 6, 8, and 10 mg/mL. For 12 and 14 mg/mL, the growth was further reduced by one order of magnitude. For the second group of experiments, the sonicated ZnO-UVM-7 suspensions progressively reduced the *E. coli* growth to reach a 4 log CFU/mL reduction for the highest concentration of antibacterial NPs. When the ZnO-UVM-7 suspensions were autoclaved before their contamination, 2 mg/mL of this material was able to provide over 5 logs CFU/mL reduction of the bacterial growth, and a concentration of 4 mg/mL was able to inhibit it completely. When the suspensions were autoclaved and sonicated, no growth was recorded for all the concentrations established during these experiments, meaning that under these conditions the *E. coli* growth can be inhibited entirely by a concentration of the ZnO-UVM-7 material lower than 2 mg/mL.

### 2.2 MICs and MBCs of the Zinc Oxide Suspensions

The MICs and MBCs of ZnO and ZnO-UVM-7 (Si/Zn = 5) suspensions, with concentration ranging from 2 to 14 mg/mL, were determined by the CFU method, after 24 h incubation at 37 °C in LB, against *E. coli* (DH5α) bacteria for different treatments.

Table 1 reports the MICs and MBCs of ZnO and ZnO-UVM-7 suspensions against *E. coli*. MICs, and particularly MBCs, were necessary to determine the minimum concentrations of antibacterial nanoparticles that would ensure the antibacterial efficacy of PLA nanofibers. The results indicated that ZnO and ZnO-UVM-7 nanoparticles with an average diameter of 20 and 5 nm, respectively, significantly inhibited (MIC) or killed (MBC) the tested bacteria. The results also demonstrate, that the combination of sterilization and ultrasound stimulations decreased considerably the MBC of ZnO and ZnO-UVM-7 nanoparticles from 14 mg/mL to 6 and 2 mg/mL, respectively.

### 2.3. Fiber Porosity Measurement

Table 2 presents the porosities of the PLA scaffolds that have been produced by the electrospinning process with the parameters mentioned in the experimental section. The results show that neat PLA mats are characterized by a porosity of about 90% that is slightly higher than that of the nanocomposite mats filled with either ZnO or ZnO-UVM-7 nanoparticles. The scaffolds that contain the ZnO-NPs provided an overall porosity around 87%, which is slightly higher than the porosity of the nanocomposites mats filled with ZnO-UVM-7.

### 2.4. Thermogravimetric Analysis (TGA)

The TGA was performed to estimate the content of the nanoparticles inside the scaffolds that have been developed by the electrospinning process. This amount was then compared to the initial weight of the nanoparticles that have been introduced, for each concentration, during the solutions’ preparations. The results are presented in Table 3. For the lowest content of the ZnO-NPs, 79% of the initial amount of the ZnO-NPs was found in the mat containing 0.6 wt % ZnO. For the mat with 0.8 wt % of ZnO, this percentage was around 63%. However, for higher ZnO contents, this rate dropped dramatically to reach 21% for the mat containing 1 wt % of ZnO.

### 2.5. Scanning Electron Microscopy (SEM)

**Figure 3:**
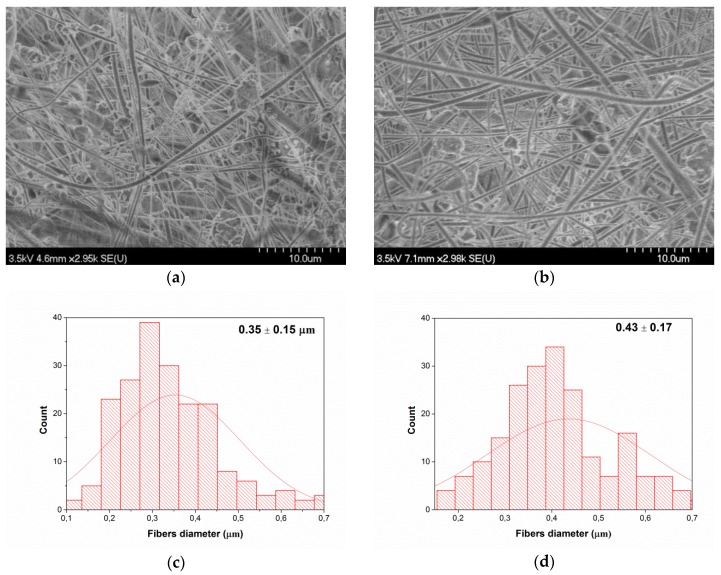
Morphology of electrospun PLA based mats: (**a**) PLA (10% *w*/*v*), 1 wt % ZnO in TFE (0.5 mL/h, 35 KV, 15 cm), (**b**) PLA (10% *w*/*v*), 1 wt % ZnO-UVM7 (Si/Zn = 5) in TFE (0.5 mL/h, 35 KV, 15 cm) and (**c**) and (**d**) are fiber diameter distribution of (**a**) and (**b**), respectively. The scale bars represent 10 μm.

In order to understand the relationship between PLA based-mat morphology and their AMA, SEM imaging was performed on the mats. In this regard, Figure 3 shows the images of PLA nanocomposite mats obtained by electrospinning under the following conditions: a feed rate of 0.5 mL/h, a voltage of 35 KV, and a needle-collector distance of 15 cm. These SEM images (Figure 3a,b) reveal the presence of a network of randomly-oriented smooth nanofibers, with some beaded-fibers, which is mostly attributed to the relatively low PLA concentration in the electrospinning solutions. Figure 3c,d show the average fiber diameters of mats obtained for PLA/ZnO and PLA/ZnO-UVM-7 solutions, respectively. The average fiber diameters were estimated from the SEM images and were 0.35 μm and 0.45 μm for PLA/ZnO and PLA/ZnO-UVM-7 nanofibers, respectively, while the fiber diameter distribution ranged from 0.1 to 0.7 μm. Both mats presented nanoparticles agglomerates in the fiber's network. Smaller aggregates formed on the surface of nanofibers, whereas the larger ones were scattered all over the fiber's network. Using Image-Pro Plus software, the average diameter of the nanoparticles agglomerates formed was estimated to be 1.4 μm.

### 2.6. ABA of PLA-Based Nanofibers

**Figure 4:**
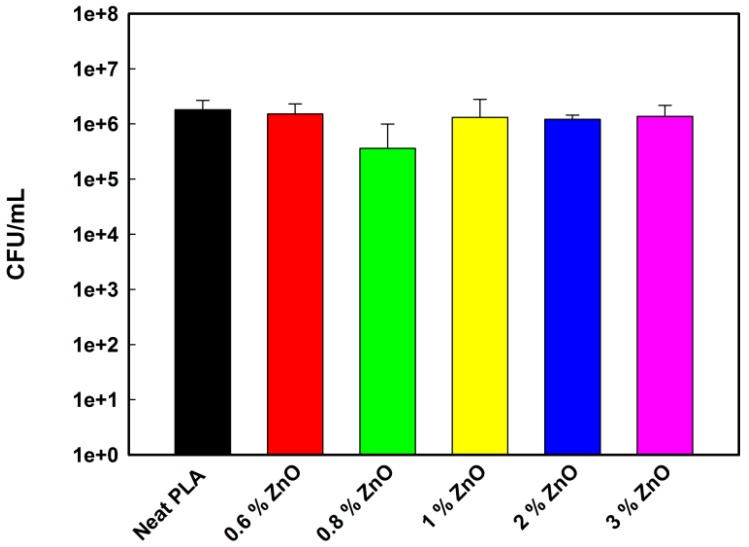
ABA of electrospun PLA/ZnO nanofibers with different ZnO content, against *E. coli* (DH5α), after incubation at 37 °C for 4 h in PBS solutions. Data are shown with mean values and standard errors of bacterial counts.

**Figure 5:**
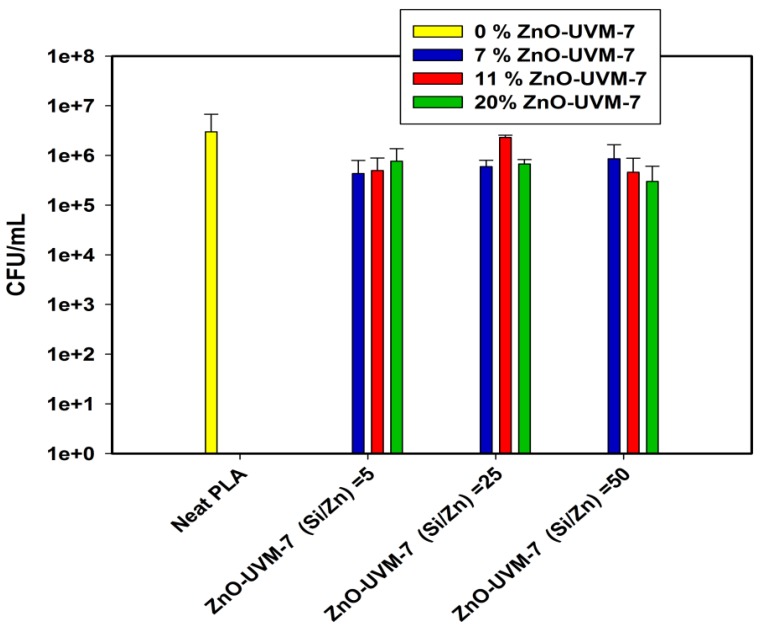
ABA of electrospun PLA/ZnO-UVM-7 nanofibers with different ZnO-UVM-7 content, for 3 different molar ratio (5, 25, and 25), against *E. coli (DH5α),* after incubation at 37 °C for 4 h in phosphate-buffered saline solutions. Data are shown with mean values and standard errors of bacterial counts.

The ABA of the ZnO and ZnO-UVM-7 nanoparticle-filled PLA fiber mats was assessed using an American Standard test method (ASTM E-2149-13a, 2013) against Gram-negative bacteria (*E. coli*). The activity of the neat PLA mats against these bacteria was used as a control. The results of the ABA analysis are shown in Figure 4 and Figure 5. After 4 h contact at 37 °C in PBS, PLA/ZnO nanofibers (Figure 4) did not show any significant reduction rate of bacterial growth of *E. coli*. The lowest content of ZnO (0.6 wt %) does not decrease the *E. coli* population growth. At 0.8 wt %, the bacterial population was reduced by less than 1 log magnitude. However, a further increase in ZnO-NPs had no significant effect, and the *E. coli* growth was in the same range as the control growth, showing the absence of ABA in these conditions.

Figure 5 shows the ABA of PLA mats containing ZnO-UVM-7 material against *E. coli* (DH5α). ZnO-UVM-7 with a molar ratio of 5 has the highest Zn content among the silica-doped nanoparticles. The increase in the concentration of this material in the precursor solutions for electrospinning decreased the growth inhibition rate from 85.5% to 74% for 7 and 20 wt % of the ZnO-UVM-7 nanoparticles, respectively. The decrease of the growth inhibition rate can be explained by the increase of the probability of the nanoparticles agglomeration. For the material having a molar ratio of 25, the PLA mats with 7 wt % exhibited an inhibition of *E. coli* growth of 80%, but this rate dropped dramatically to 23% for a content of 11 wt %, and then increased again to 77% for the highest concentration of ZnO-UVM-7 nanoparticles. For a molar ratio of 50, the increase in the content of the ZnO-UVM-7 nanoparticles generated an increase in the growth inhibition rate up to 90% for 20 wt % ZnO-UVM-7 nanoparticles. This trend was only observed for the material having the lowest content in zinc.

## 3. Discussion

Many studies have strongly indicated that ZnO-NPs have a concentration-dependent ABA [9,10,12,25,27,28,29,30,31,32]. Jalal and co-workers [27] indicated that the increase of nanoparticle concentration produced higher ABA towards *E. coli* due to the increase of the amount of H_2_O_2_ generated from the surface of ZnO-NPs [27]. Sawai and co-workers [28] suggested that H_2_O_2_ is one of the primary factors in the antibacterial mechanism of the ZnO powder slurry. Since the bacterial cell membrane is relatively permeable to these species, it is assumed that they are able to penetrate the cell membrane of *E. coli*, produce injury, and inhibit cell growth [28]. In a previous study [29], they measured the ROS generated in ZnO slurries by chemiluminescence and oxygen electrochemical analyses and found that H_2_O_2_ produced a concentration that was linearly proportional to the ZnO concentrations [29]. Moreover, the ultrasound stimulations can have an enhancement effect on reducing the *E. coli* growth. This enhancement can be due to nanoparticle dissociation, which will increase nanoparticles penetration into cell membranes in the presence of ultrasound treatment. Additionally, the sterilization of the NP suspensions played a crucial role on the enhancement of the ABA of ZnO-NPs by removing the contaminants and naturally-occurring micro-organisms that already exist in suspensions before their inoculation. In the case of the last group of experiments, the enhancement in the ABA was due to the combination of the autoclaving and the ultrasound effect with the antibacterial effect of the ZnO-NPs. 

To the best of our knowledge, the combinatory antibacterial effect of autoclaving, ultrasound stimulations, and ZnO-NPs on *E. coli* has not been studied previously. Thus, the objective of these experiments was to determine the combined influence of ultrasound and autoclave sterilization on inhibiting *E. coli* functions by the ZnO-NPs. The only existing study that has focused on the combined antibacterial effect of ZnO-NPs suspensions has investigated the antibacterial effect of ZnO-NPs in both the absence and the presence of ultrasound stimulations [39]. They concluded that any increase in the antibacterial activity of ZnO-NPs (20 nm), in the presence of ultrasound stimulations, must be attributed to a synergistic antibacterial mechanism of the combination of the nanoparticles and ultrasound. They assume that the presence of ultrasound stimulations can have a significant effect on decreasing bacteria functions. They explained that, in the presence of ultrasound, the agglomerated nanoparticles with negative surface charge are dissociated, and the flocculated bacteria may also be disrupted. Thus, physical interactions between the nanoparticles and the bacteria membrane may be enhanced: the transport of nanoparticles to the bacteria and their penetration across the bacteria membranes are promoted. Furthermore, the enhanced antibacterial activity observed may be due to the increased zinc ions Zn^2+^ present in the NPs suspensions after sonication, since ultrasonication is a method frequently used to improve the solubility of nanomaterials [39].

As discussed above, the combined effect of sterilization, ultrasound stimulations, and ZnO-UVM-7 material has a significant effect on increasing the ABA of this material. In the presence of sonication and ultrasound stimulations, a complete inhibition of microbial growth was achieved at a level of 6 mg/mL of ZnO, whereas this level was 2 mg/mL for the ZnO-UVM-7 material. This was expected since the average diameter of the ZnO-UVM-7 material (5 nm) is much smaller than the average diameter of ZnO-NPs (20 nm). In addition to the concentration-dependent ABA of ZnO-NPs that has been already discussed above, many studies strongly indicated that ZnO-NPs have a particle size-dependent ABA [7,9,10,24,25,26]. In fact, one of the parameters involved in ZnO-NPs antibacterial properties is the specific surface area. It increases for reduced particle size, thus enhancing particle surface reactivity [9,10,24,25]. Moreover, generation of H_2_O_2_ depends mainly on the surface area of ZnO-NPs [24,42]. Padmavathy and Vijayaraghavan [24] studied the AMA of ZnO suspensions against *E. coli* with various particle sizes (12 nm, 45 nm, and 2 μm), using a standard microbial method. They concluded that using small nanoparticles, thus having a larger surface area, will generate a higher concentration of ROS on the surface and will result in greater AMA. More precisely, in the case of smaller ZnO-NPs, more particles are required to cover a bacterial colony (2 μm), which results in the generation of a larger number of ROS, killing bacteria more effectively [24]. Yamamoto [10] used electrical conductivity to evaluate the effect of primary particle size on the ABA of ZnO-NPs (100–800 nm) obtained by milling in a planetary ball mill. He showed that the ABA increased with decreasing particle size [10].

Many factors can explain that the combination of sterilization and ultrasound stimulations decreased considerably the MBC of ZnO and ZnO-UVM-7. In fact, autoclaving the glass powder vials directly before their inoculation with *E. coli* can allow the removal of any contaminants and the naturally-existing micro-organisms in the antibacterial powder used or the LB medium prepared in the laboratory. On the other hand, the antibacterial mechanisms of nanoparticles may be enhanced in the presence of ultrasound stimulations. In fact, physical interactions between nanoparticles and bacteria membrane may be reinforced due to nanoparticles dissociation and increased nanoparticles penetration into the cell membrane. Moreover, antibacterial metal ions may be released from the particles’ surface more easily during the ultrasound to inhibit bacteria proliferation. Only a few studies performed on the MIC and MBC of ZnO-NPs were available in the literature. Reddy and co-workers [37] have found that *E. coli* growth was completely inhibited at a concentration of 3.4 mg/mL of ZnO-NPs with an average diameter of 13 nm. In another study, Emami-Karvani and Chehrazi [40] evaluated the MIC and the MBC of ZnO-NPs with an average diameter of 3 nm by agar diffusion testing. They found that the concentration that prevented *E. coli* growth was 1 mg/mL whereas the MBC was around 16 mg/mL. A comparison between this study and those presented above is quite complicated since the ZnO-NPs that have been used did not have the same average diameter and, in each case, different antibacterial methods were used to evaluate MIC and MBC. 

The TGA results showed that ZnO-NPs loss increased with the increase of its content. This loss is mainly due to agglomeration and sedimentation in solution. Indeed, the solutions for electrospinning were sonicated before the electrospinning to dissociate the nanoparticles in solution, but this process is performed under static conditions over several hours. During the electrospinning process, a certain amount of nanoparticles will sediment in the bottom of the syringe, depending on the ZnO concentration, and are not ejected with the solution.

Regarding the SEM observations, similar results were obtained by Rodriguez-Tobias and co-workers [22] who produced PLA-based mats containing ZnO-NPs with an average diameter of 12 nm, synthesized by a microwave-assisted technique. Their mats were produced from TFE solutions with 10% (*w*/*v*) of PLA at a voltage of 25 KV, a feed rate of 2.5 mL/min, a rotating collector speed of 700 rpm, and a needle-to-collector distance of 25 cm. The nanofibers were characterized by an average diameter of 700-800 nm, and by the presence of some aggregates up to 300 nm within the PLA fibers [22]. Concerning the absence of the ABA of the PLA-based nanofibers at a content of ZnO (0.6 wt %), it has already been shown that, at very low concentration, ZnO-NPs are non-toxic for *E. coli* since the bacteria can metabolize Zn^2+^ as an oligo-element [34]. In fact, Reddy and co-workers [37] showed that a concentration of 1 mM of ZnO-NPs was able to consistently increase the number of CFU of *E. coli* compared to the control, whereas the growth of *S. aureus* was completely inhibited at a concentration ≥1 mM [37]. In another study conducted by Padmavathy and Vijayaraghavan [24], suspensions at low concentrations of ZnO-NPs (0.01–1 mM) had a slight AMA against *E. coli*, the presence of soluble Zn^2+^ acted as nutrients for this bacteria since the zinc element is an essential cofactor in a variety of cellular processes [15,24]. The slight antibacterial performance provided for 0.8 wt % ZnO-NPs, could be associated with a higher surface area of nanoparticles subject to attack the bacteria by the well-known antibacterial mechanism for metal oxides [7,9,24,27,28,34,36,37]. The further increase in ZnO concentration provoked agglomeration of nanoparticles as it was evidenced by SEM and TGA. In fact, according to the TGA results, the increase of the ZnO-NPs content from 0.8 to 1 wt %, decreased dramatically the ZnO-NPs amount in the PLA-based mats from 63% to 21.5% compared to the initial amount of nanoparticles used for the preparation of the precursory solutions for electrospinning, and this amount was kept in the range of 22–28% for the highest concentration. Thus, for 1–3 wt %, a negligible amount of agglomerated ZnO-NPs was incorporated inside PLA-based mats, reducing the ZnO surface area and consequently, decreasing the antibacterial performance. Rodriguez-Tobias and coworkers [22] tested the AMA of PLA-based mats derived from the electrospinning process containing pure ZnO-NPs. They found that 1 wt % of ZnO-NPs demonstrated a growth inhibition rate of only 9.6%. This reduction was 25.8% for a 3 wt %, but then was lowered to 14.6% when the content reached 5 wt % of the ZnO-NPs because of nanoparticles agglomerations [22]. In the present study, the growth inhibition rate was around 17% for 0.6 wt % ZnO-NPs, which increased dramatically to reach 80% at 0.8 wt %, and finally dropped in the range 28–33% for the highest content. Thus, depending on the conditions used during the electrospinning process and the average diameter of the ZnO-NPs used, there is an optimum ZnO-NPs concentration characterized by a higher growth inhibition rate, above this concentration, a drop in the inhibition rate will be observed because of the high probability of the nanoparticle agglomeration.

Furthermore, it can be concluded that *E. coli* exhibited a low sensitivity to the polymer mats with different content of ZnO-NPs. Such results were confirmed by several studies [22,23,34,43]. For example, Rodriguez-Tobias and co-workers [22,23] confirmed this result through two different studies, and noticed that simultaneous electrospinning of PLA solutions with electrospraying of ZnO dispersions, as well as the use of ZnO-graft-PLA nanoparticles, provided better antibacterial performance than the PLA-based mats filled with pure ZnO-NPs and developed by the electrospinning process [22,23]. The low sensitivity of *E. coli* to polymer-based ZnO-NPs was explained by the complex cell wall structure of Gram-negative bacteria with a thin layer of peptidoglycan between the outer membrane and cytoplasmic one, and the overall negative charge of the bacterial cell surface. In addition, in the present study, the ester groups in PLA mats increase the electron density. Hence, electrostatic repulsion between PLA nanofibers and the partial negatively-charged *E.coli* cell surface are likely [43]. The ABA of nanosized silicon dioxide (SiO_2_) has been performed by Adams and co-workers [35] in water suspensions. It was observed that these SiO_2_ nanoparticle suspensions (205 nm) led to less than 20% growth inhibition against *E. coli*, whereas ZnO-NPs (480 nm) showed around 50% of growth inhibition under the same conditions. It was also noticed that light had no significant effect on increasing the toxicity of SiO_2_ [35]. These results were in disagreement with those obtained by Liang and co-workers [44] who found that micro-sized bulk SiO_2_ was inert towards bacteria [44]. In a more recent study, a SiO_2_/ZnO composite material obtained by two synthesis routes—a sol-gel followed by alkaline precipitation and acidic precipitation—demonstrated its effectiveness in inhibiting *E. coli* growth with inhibition growth ranging from 75% to 83% [45]. This work confirmed the findings of Adams and co-workers [35] that showed that the antibacterial activity of pure ZnO-NPs had a higher activity than the SiO_2_/ZnO composite material [35,45]. The growth inhibition rates obtained for PLA mats containing ZnO-UVM-7 material in the present work are in agreement with those found by Spoiala’s group [45], with an inhibition rate ranging from 70% to 90%.

Comparing ZnO-NPs/PLA-based mats to those from PLA/ZnO-UVM-7, it is possible to affirm that it is possible to maintain a growth inhibition rate above 70% by using ZnO-UVM-7. This was probably due to the reduced quantity of agglomerated nanoparticles. The incorporation of metal to the silica system by a direct and reproducible one-pot surfactant-assisted procedure allowed the production of silica-based material with ZnO-NPs diameters ranging from 3 to 4 nm. The use of the silica-doped nanoparticles contributed to reduce the agglomeration and at the same time to use antibacterial nanoparticles with very low diameter, providing a higher surface area. 

The generation of ROS depends on the surface area of nanoparticles, i.e., the amount of H_2_O_2_ generated should increase with the concentration and surface area of ZnO-NPs [7,24]. Since the hydroxyl radicals and superoxide are negatively-charged particles, they cannot penetrate the cell membrane and have to remain in direct contact with the outer surface of the bacteria. However H_2_O_2_ can easily penetrate the cell [24,27]. In fact, the generated ROS can cause the oxidation of the lipid membrane in the cell wall of *E. coli*. In some studies, it was demonstrated that the ABA of ZnO-NPs could occur even in the dark [35,46], indicating that there are other mechanisms apart from those that require UV irradiation for the production of ROS in the absence of light. The negatively-charged bacterial cell [11] will allow strong electrostatic binding between the nanoparticles and the bacteria surface [36], thus producing cell membrane damage [9] that will lead to the leakage of intracellular content [38]. In fact, the ZnO-NPs can either be transported into the cytoplasm or penetrate the cell wall [26,34,47].

The non-toxicity of the electrospun mats produced with the same setup was already assessed by a large number of researchers in our laboratory. It was proven that no residual solvent was remaining in the nanofiber mats. Moreover, most of these nanofibers served in biomedical applications, which could prove their non-toxicity even more. PLLA electrospun nanofibers were produced from TFE solutions. Neural stem-like cells (NSLCs) cultured on these mats have successfully proliferated and differentiated rapidly into motor neurons. These structures may be able to offer a support for the fabrication of motor nerve grafts from NSLCs [48]. In another study, PLA electrospun nanofibers were developed from a mixture of dichloromethane (DCM) and trifluoroacetic acid (TFA). The PLA mats also supported neural stem cell (NSC) adhesion and proliferation, and were, thus, used in developing efficient approaches for NSC expansion in neural tissue engineering applications [49]. In addition, the antibacterial test for the nanofiber mats were performed in PBS solution, which is a water-based medium. Thus, if there was residual solvent remaining inside the nanofiber mats, they would dissolve when immersed in PBS solution. However, on the contrary, they remained intact after four hours of incubation.

## 4. Materials and Methods

### 4.1. Materials

PLA was a semi-crystalline grade (INGEO^TM^ Biopolymer 4032D) from NatureWorks LLC (Blair, NE, USA), with M_n_ = 133,000 and molecular weight distribution M_w_/M_n_ =1.9. Other characteristics from the manufacturer were: relative viscosity of 3.94, D stereo-isomer content of 1.4%, and residual monomer content of 0.14%. Commercially-available ZnO-NPs were kindly supplied by SkySpring Nanomaterials Inc (Houston, TX, USA) and were used without further purification. The average diameter specified by the supplier was 20 nm, and the specific surface area 30 to 50 m^2^/g. A second zinc oxide-based nanoparticle system was used and consisted in ZnO nanodomains embedded in bimodal mesoporous silica. This composite powder, referred to as ZnO-UVM-7, was used with three different Zn content (Si/Zn = 5, 25, and 50), and was produced by the atrane method [50,51,52]. The final ZnO-UVM-7 material was bimodal and mesoporous with high surface area up to 1000 m^2^/g. These nanoparticles had a diameter ranging from 2 to 5 nm [50]. TFE (TFE ≥ 99%) was purchased from Sigma-Aldrich Co.LLC (Oakville, ON, Canada). Cultures of *Escherichia coli* (*E. coli* strain *DH5α* non-pathogen) were obtained from the Laboratory of Microbiology, Infectiology and Immunology (Université de Montréal, Québec, Canada).

### 4.2. Solutions Preparation

In this work, two different solution systems were prepared to investigate the effect of nanoparticles content. In the first one, 0, 0.6, 0.8, 1, 2, and 3 wt % of ZnO-NPs, with respect to polymer, were dispersed in 10% PLA (*w*/*v*) solutions in TFE. In the second, each of the three ZnO-UVM-7 powders was dispersed in PLA/TFE solutions at concentrations of 7, 1, and 20 wt % with respect to polymer. Solutions mixing was performed at room temperature using a laboratory magnetic stirrer (Corning Inc, Tewksbury, MA, USA) for 24 h to ensure complete dissolution of the polymer and to obtain homogenous solution with a good dispersion of the nanoparticles.

### 4.3. Electrospinning Process

Electrospinning was performed at room temperature using a homemade horizontal setup. Each of the prepared PLA solutions was contained in a 10 mL plastic syringe, connected to a stainless steel needle (OD = 18 gauge), and placed in a programmable micro-syringe pump (Havard Apparatus, PHD2000, Havard Apparatus, Holliston, MA, USA). The delivery rate of the spinning solution was 0.5 and 1 mL/h for the PLA solutions prepared in the presence of ZnO-NPs and ZnO-UVM-7, respectively. The electrospinning was conducted at a tip-to-collector distance of 15 cm. A variable voltage of 30–35 KV was provided using a common high-DC voltage power supply (ES60P-5W Gamma High Voltage Research Inc., Ormond Beach, FL, USA). A web of fibers was collected on a grounded stationary collector covered with aluminum foil. All experiments were conducted in a chamber at a relative humidity of 20–30% and under atmospheric pressure. Collected electrospun fibers were dried overnight under a chemical fume hood for the evaporation of any remaining solvent.

### 4.4. Scanning Electron Microscopy (SEM)

The morphology of electrospun PLA nanofibers was observed with a JSM-7600TFE field emission gun-scanning electron microscope (FEG-SEM) (HITACHI, Calgary, AB, Canada) operating at 5–10 KV. For better conductivity and to reduce electron charging effects, samples were observed as collected on aluminum foil after sputter-coating with gold. The fiber diameter and fiber diameter distribution were analyzed using Image-Pro Plus software. Approximately, 600 nanofibers randomly chosen from three independent samples (200 nanofibers from each sample) were used for the analysis.

### 4.5. Thermogravimetric Analysis (TGA)

The amount of zinc oxide powder inside the PLA nanofibers was determined by using a Q500 TGA system (TA Instruments, New-Castle, DE, USA). The measurements were performed under nitrogen (N_2_) atmosphere from 25 to 800 °C at a rate of 10 °C/min.

### 4.6. Fiber's Porosity Measurement

The porosity of the nanofiber scaffolds was measured using the liquid intrusion method. Scaffolds were weighed prior to their immersion in ethyl alcohol, and they were left overnight on a shaker table to allow diffusion of ethanol into the void volume. The next day, the scaffolds were taken out, blotted with a kimwipe to remove the excess ethanol, and reweighed. This measurement was performed in duplicate samples. The porosity was calculated by dividing the volume of intruded ethanol (as determined by the change in mass due to intrusion and the density of ethanol, 0.789 g/mL) by the total volume after intrusion (i.e., the volume of the intruded ethanol combined with the volume of the PLA fibers determined from the initial mass of the PLA scaffold and the density of PLA, 1.25 g/cm^3^) [53,54].

### 4.7. Antibacterial Test

#### 4.7.1. Effect of the Combined Effect of Sterilization and Ultrasound Stimulations on the ABA of the Zinc Oxide Suspensions

Bacteria were grown in a nutritional-rich medium (Luria Bertani, or LB, broth) under constant agitation for 24 h at 37 °C until reaching a density of approximately 10^9^ colony forming units (CFU/mL). In the testing tubes, different concentrations of the analyzed powder were prepared in the presence of LB broth. Then, the appropriate volume of *E. coli* (DH5α) inoculum was added to reach a bacterial concentration of 10^5^ CFU/mL, and the tubes were incubated at 37 °C with shaking for 24 h. The day after, for each sample, six serial dilutions of phosphate-buffered saline (PBS) solution were prepared. Three 10 μL droplets were taken from each of the six dilutions and were applied onto the LB agar plate. This was also performed for the control tubes, which contained only bacterial culture (i.e., without antibacterial powder). Four different groups of experiments were conducted: in the first one; the powder suspensions were tested directly after their preparation referred as, −S, −U. In the second one, the suspensions were subjected to ultrasonic stimulations (−S, +U). In the third one, the suspensions were autoclaved to eliminate naturally-occurring micro-organisms and used immediately for the following antibacterial tests (+S, −U). In the fourth group, powder suspensions were subjected to both ultrasound stimulations and autoclaving before their contamination (+S, +U). All the tests were performed in triplicate, and the results were expressed as mean values. The minimum concentrations of antibacterial nanoparticles necessary to inhibit bacterial growth (MIC) and to kill bacteria (MBC) were also determined by the CFU method (ASTM E-2149-13a, 2013), one of the most commonly used techniques for the enumeration of bacteria. 

#### 4.7.2. In Vitro Antibacterial Efficiency of Nanocomposite PLA Nanofibers

The antibacterial properties of the different PLA nanocomposite mats were evaluated by using a non-pathogen *E. coli* (DH5α) as a model bacterium and by following The American Society for Testing and Materials (ASTM E-2149-13a, 2013) standard for antimicrobial agents. Bacteria were grown in a rich medium (LB broth) under constant agitation for 24 h at 37 °C until reaching a density of approximately 10^9^ colony forming units (CFU)/mL. Then, the bacterial culture was diluted in a buffer, a non-permissive growth condition (phosphate-buffered saline, or PBS, solution), in order to have a density of approximately 10^6^ CFU/mL. The PLA nanofiber (4 cm^2^) mats were immersed (after sterilization under UV light for 20 min) into 5 mL of the PBS culture medium containing *E.* coli. Autoclaving was not the suitable method for fiber sterilization as the samples will not be recoverable after immersing them in water. Moreover, the properties of the PLA mats may change at high temperature, since the autoclaving temperature is higher than the glass transition temperature of PLA. Neat PLA fiber mats and untreated bacteria were suspended in PBS and prepared in the same conditions to be used as positive and negative controls, respectively. Then, tubes were incubated at 37 °C (the optimal temperature for bacterial growth) for 4 h in an orbital shaker (New Brunswick). Dilutions of the inoculated suspensions were prepared and deposited on LB agar plates and incubated overnight (18 h) at 37 °C for the counting of the surviving bacteria (CFU/mL). All experiments were carried out in triplicate.

All the antimicrobial tests were conducted in the Department of Microbiology, Infectiology, and Immunology at Université de Montréal.

## 5. Conclusions

In this study, the ABA of suspensions of pure ZnO nanoparticles (ZnO-NPs) and mesoporous silica doped with ZnO (ZnO-UVM7), as well as electrospun nanofibers containing those nanoparticles, were investigated. The antibacterial results showed that the combination of ZnO and ZnO-UVM-7 suspensions with pre-treatments, such as ultrasound stimulations and sterilization, could help to enhance their ABA against *E. coli.* The use of these pre-treatments also decreased the MBC of the powders’ suspensions considerably. Thus the bacteriostatic/bacteriocidal effect of these suspensions has occurred at a lower content of nanoparticles. The *E. coli* growth was completely inhibited at a concentration of 6 and 2 mg/mL for ZnO and ZnO-UVM-7, respectively, in the presence of theses pre-treatments. Thereby, the use of mesoporous silica doped with ZnO has allowed the improvement of the ABA of these suspensions. The ABA results have confirmed that the zinc oxide suspensions have a concentration-dependent and size-dependent activity.

Electrospinning was used to fabricate nanofiber mats of PLA filled with either ZnO or ZnO-UVM-7 nanoparticles. SEM images showed the presence of nanoparticle agglomerates of around 1 μm in diameter within the nanofiber's network. The absence of the ABA of PLA-based nanofibers at the lowest content (0.6 wt %), was explained by the fact that, at very low concentration, ZnO-NPs are non-toxic for *E. coli* since they can metabolize Zn^2+^ as an oligo-element. The increase in ZnO content above 1 wt % increased the probability of agglomeration and sedimentation and dramatically reduced the ZnO-NPs amount in the PLA-based mats. Thus, the growth inhibition rate of *E. coli* was reduced considerably from 80% to 30% for the highest ZnO content. The use of ZnO-UVM-7 silica-doped nanoparticles had probably improved the nanoparticles’ dispersion since the PLA nanofibers filled with ZnO-UVM-7 were able to maintain an inhibition growth rate higher than 70%.

## Figures and Tables

**Table 1:** MICs and MBCs of ZnO and ZnO-UVM-7 (Si/Zn = 5) suspensions in mg/mL, after 24 h incubation at 37 °C in LB, against *E. coli* (DH5α) bacteria for different treatments.

Treatment Antibacterial NPs	−S,−U		−S,+U		+S,−U		+S,+U	
	MIC	MBC	MIC	MBC	MIC	MBC	MIC	MBC
**ZnO**	2	>14	2	>14	2	8	2	6
**ZnO-UVM-7**	2	>14	2	>14	2	4	<2	2

**Table 2:** Porosity of PLA nanofiber scaffolds produced by the electrospinning process, measured by the liquid intrusion method at room temperature.

Samples	Porosity ɛ (%)
Neat PLA nanofibers	90.08
PLA/0.8 wt % ZnO nanofibers	87.48
PLA/3 wt % ZnO nanofibers	87.07
PLA/0.6 wt % ZnO-UVM-7 (Si/Zn = 5) nanofibers	86.95
PLA/0.8 wt % ZnO-UVM-7 (Si/Zn = 5) nanofibers	84.59

**Table 3:** Estimation of ZnO-NPs amount in PLA mats produced by the electrospinning process, by using TGA under nitrogen (N_2_) atmosphere from 25 to 800 °C, at a rate of 10 °C/min

ZnO Content	ZnO Weight in Solutions (mg)	ZnO Weight in PLA Nanocomposites Mats (mg)	Percentage of ZnO in the PLA Nanocomposite Mats (%) (Compared to 100% if No Loss)
PLA/0.6 wt % ZnO	12	9.49	79.08
PLA/0.8 wt % ZnO	16	10.13	63.31
PLA/1 wt % ZnO	20	4.31	21.55
PLA/2 wt % ZnO	24	5.48	22.83
PLA/3 wt % ZnO	28	8.03	28.67
